# A Biomimetic Membrane Device That Modulates the Excessive Inflammatory Response to Sepsis

**DOI:** 10.1371/journal.pone.0018584

**Published:** 2011-04-14

**Authors:** Feng Ding, Joon Ho Song, Ju Young Jung, Liandi Lou, Min Wang, Linda Charles, Angela Westover, Peter L. Smith, Christopher J. Pino, Deborah A. Buffington, H. David Humes

**Affiliations:** 1 Division of Nephrology, Huashan Hospital, Fudan University, Shanghai, China; 2 Department of Internal Medicine, Center for Advanced Medical Education by BK21 Project, Inha University School of Medicine, Incheon, Korea; 3 Chungnam National University, Daejoen, Korea; 4 Innovative BioTherapies, Inc., Ann Arbor, Michigan, United States of America; 5 Department of Internal Medicine, University of Michigan, Ann Arbor, Michigan, United States of America; University of Pennsylvania, United States of America

## Abstract

**Objective:**

Septic shock has a clinical mortality rate approaching fifty percent. The major clinical manifestations of sepsis are due to the dysregulation of the host's response to infection rather than the direct consequences of the invading pathogen. Central to this initial immunologic response is the activation of leukocytes and microvascular endothelium resulting in cardiovascular instability, lung injury and renal dysfunction. Due to the primary role of leukocyte activation in the sepsis syndrome, a synthetic biomimetic membrane, called a selective cytopheretic device (SCD), was developed to bind activated leukocytes. The incorporation of the SCD along an extracorporeal blood circuit coupled with regional anticoagulation with citrate to lower blood ionized calcium was devised to modulate leukocyte activation in sepsis.

**Design:**

Laboratory investigation.

**Setting:**

University of Michigan Medical School.

**Subjects:**

Pigs weighing 30-35 kg.

**Interventions:**

To assess the effect of the SCD in septic shock, pigs were administered 30×10^10^ bacteria/kg body weight of *Escherichia coli* into the peritoneal cavity and within 1 hr were immediately placed in an extracorporeal circuit containing SCD.

**Measurements and Main Results:**

In this animal model, the SCD with citrate compared to control groups without the SCD or with heparin anticoagulation ameliorated the cardiovascular instability and lung sequestration of activated leukocytes, reduced renal dysfunction and improved survival time compared to various control groups. This effect was associated with minimal elevations of systemic circulating neutrophil activation.

**Conclusions:**

These preclinical studies along with two favorable exploratory clinical trials form the basis of an FDA-approved investigational device exemption for a pivotal multicenter, randomized control trial currently underway.

## Introduction

The sepsis syndrome is defined as the systemic inflammatory response to infection. Sepsis is the leading cause of death in critically ill patients in the United States, affecting 750,000 people annually [1], [2]. Despite prompt treatment with antibiotics, fluid resuscitation and artificial organ function support, mortality rates still exceed 30 percent [3], [4]. Most infections are bacterial, and, as sepsis progresses in severity, the patient develops cardiovascular instability with hypotension, lung dysfunction, and renal function deterioration [5], [6]. These major clinical manifestations of sepsis, however, are not caused directly by the invading microbes but are results of dysregulation of the patient's own inflammatory response [5], [7]. Central to the initial innate immunologic response to infection is the leukocyte, especially the neutrophil [6], [8]. The neutrophil is a short-lived circulating phagocyte which, when activated, binds to the microvascular endothelium and extravasates into local tissue spaces to degrade injured tissue or kill ingested pathogens with a variety of stored proteolytic enzymes and rapid production of reactive oxygen species [6], [8]. Its essential role in sepsis is demonstrated by the recurrence of life-threatening infections in patients with neutropenia or with leukocyte defects [9], [10].

Although critical in host defense, the activation of circulating neutrophils and the microvascular endothelium in systemic infections are the basis for the progression to multiorgan dysfunction in severe sepsis [6], [7]. The interaction of activated neutrophils and endothelium leads to increased vascular permeability with fluid leakage from the intravascular space to tissue interstitium with resulting hypovolemia, hypotension and cardiovascular instability [7], [11]. Sequestration and aggregation of neutrophils in the peritubular capillaries of the kidney promotes acute kidney injury (AKI) and, if substantive, acute renal failure (ARF) [12], [13]. Sequestration and infiltration in lung tissue progresses to diminish pulmonary gas exchange and, if severe, adult respiratory distress syndrome (ARDS) [14], [15].

New therapies directed to treating sepsis have, in the past, focused on interrupting the excessive levels of inflammatory cytokines (cytokine storm) or the activation of the coagulation system during sepsis, with little or modest effects on this disease process when tested clinically [5]. The administration of granulocyte-colony stimulating factor (G-CSF) to enhance neutrophil number during sepsis has also failed to improve clinical survival rates in several studies [16], [17].

Since activated leukocytes are central to the pathogenesis and progression of sepsis and other clinical inflammatory disorders, a variety of new therapeutic approaches are being considered to limit the deleterious clinical effect of activated leukocytes that result from a dysregulated immune response to sepsis [18]. Our group has developed a selective cytopheretic device (SCD) composed of a synthetic biomimetic membrane that binds and sequesters activated leukocytes from the systemic circulation along an extracorporeal blood circuit. The SCD incorporates a low-velocity, low–shear force blood flow path around a bundled collection of biocompatible membranes to reproduce capillary shear in order to bind activated leukocytes during a systemic inflammatory disease state. To further minimize the systemic effects of activated leukocytes, the blood is anticoagulated with regional citrate infusion to lower blood ionized calcium (iCa) levels to 0.2–0.5 mM, levels which inhibit the coagulation system of the blood. This lowering of blood iCa also has an inhibitory effect on neutrophil activation [19], thereby simultaneously combining the SCD effect to sequester activated circulating leukocytes and limit the potential activation of leukocytes entering the SCD and the low-iCa environment.

The SCD with citrate anticoagulation was evaluated in a well-established porcine model of *Escherichia coli (E. coli)*-induced septic shock [20] and demonstrated an ability to lower systemic neutrophil activation, diminish aggregation of activated leukocytes in the lungs, decrease systemic capillary leak, preserve cardiac output (CO) and mean arterial pressure, ameliorate renal dysfunction, and prolong survival time compared to various control groups. This approach is currently being tested in a pivotal multicenter, randomized, control clinical trial with an investigational device exemption (IDE) from the Food and Drug Administration (FDA).

## Methods

### Animal model

The animals were prepared for study using a protocol previously reported in detail [20]. In brief, pigs weighing 30–35 kg were anesthetized and artificially ventilated. Arterial and Swan-Ganz thermodilution catheters, which were connected to transducers, were placed to monitor cardiovascular parameters at various time intervals. An ultrasonic flow probe was placed on one renal artery for continuous assessment of renal blood flow (RBF). Septic shock was induced by infusion of 30×10^10^ bacteria/kg body weight of *Escherichia coli* into the animals' peritoneal cavities. All groups received identical volume resuscitation protocols but no vasopressor or inotropic agents as described previously [20]. Volume resuscitation consisted of 80 mL/kg crystalloid and 20 mL/kg colloid (Hespan, 6% hetastarch) as an acute bolus immediately following bacteria administration, followed by a continuous infusion of crystalloid at 300 mL/hr for 0–3 hr and 450 mL/hr for 3 hr until the end of the experiment. Ultrafiltration rate from the hemofilter was maintained at 5 mL/min in all groups. For heparin animals, volume resuscitation consisted of Na 150, Cl 115, HCO_3_ 38, Ca 2.5, and Mg 1.6 mEq/L in D_5_W at a rate of 300 mL/hr from 0–3 hr and 450 mL/hr for 3 hr until the remainder of the experiment. For citrate animals, volume resuscitation consisted of ACD-A citrate (Baxter, IL) at 100 mL/min before the hemofilter and 2% CaCl_2_ at 40 mL/min after the SCD to maintain iCa levels along the circuit and in the systemic circulation of the animal described below. The volume replacement of the citrate animals was supplemented with normal saline to maintain a total administered dose at 300 mL/hr from 0–3 hrs and 450 mL/hr from 3 hr until experiment termination. This acute model of septic shock results in death due to hypovolemia and hypotension secondary to a profound capillary leak process.

### Extracorporeal circuit of the SCD

**Figure 1:**
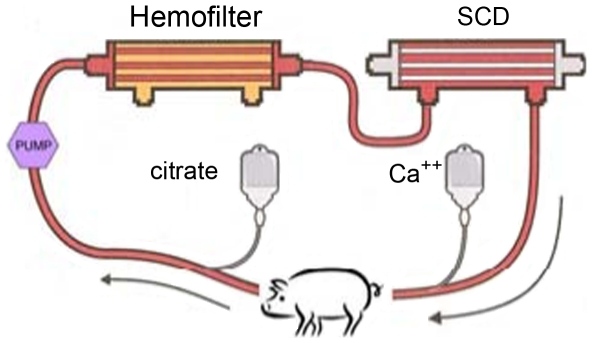
Extracorporeal circuit with SCD.

Immediately after bacteria administration, the animal was connected to an extracorporeal circuit containing a standard continuous renal replacement therapy (CRRT) hemofilter and an SCD, as displayed in Figure 1. Extracorporeal blood flow was regulated at 100–150 mL/min. The hemofilter was a conventional hemofilter (F40, Fresenius AG). The SCD was a cartridge containing polysulfone hollow fibers with special blood line connectors from the blood port of the first hemofiltration cartridge to the SCD side port. The tested SCD had either a membrane surface area of 0.7 or 1.8 m^2^ on the lumen side was supplied by CytoPherx, Inc. (Ann Arbor, MI). Extracapillary space (ECS) surface area was approximately 1.0 or 2.5 m^2^ respectively. The hollow fibers had a molecular weight cut off (MWCO) of 65 kDa and an inner diameter of 200 µm and wall thickness of 40 µm, with the 0.7 m^2^ cartridge containing roughly 4,200 fibers and the 1.8 m^2^ cartridge approximately 10,900 fibers. The lumen fill volumes of the 0.7 m^2^ cartridge were 42 mL and 110 mL for the 1.8 m^2^ cartridge, and the ECS fill volumes were 130 mL and 250 mL respectively. At the beginning of the experiment, the lumens of the fibers were filled with normal saline and the end ports capped. The dialysis pump system utilized either a Gambro AK-10 or Fresenius 2008H. The pressure drop across the SCD was 70–75 mmHg. Two additional groups of pigs were evaluated. For control-group comparisons, another group of three animals underwent extracorporeal blood perfusion in a circuit containing a hemofilter without the SCD and blood perfusion through the conventional blood flow pathway within the lumens of the hollow fibers in the hemofilter. These animals received citrate regional anticoagulation and are labeled the conventional citrate (Con-citrate) group. A final group of animals were treated similarly to the SCD group with citrate but without the bacterial infusion. This group is referred to as the non-septic control (NS-control) animals.

### Anticoagulation process

The anticoagulation process was a major variable in this series of experiments. Group SCD-heparin (H, n = 12) received standard systemic heparinization to maintain patency of the extracorporeal circuit with targeted activated clotting times (ACTs) of 200–300 sec; group SCD-citrate (C, n = 13) received regional citrate anticoagulation [21], [22], [23]. Anticoagulation citrate dextrose-A (ACD-A, Baxter) was infused pre-hemofilter at a rate of 100 mL/hr to maintain the iCa concentration in the circuit at 0.2–0.5 mmol/L. Calcium chloride (2% w/v) was infused into the venous return of the circuit at approximately 40 mL/hr to maintain systemic iCa values between 1.1 and 1.3 mmol/L. These iCa levels were monitored utilizing an iSTAT reader (Abbott Labs). An additional group of SCD-C utilized a 1.8 m^2^ SCD (n = 3) for dose comparisons to SCD-C, 0.7 m^2^ group.

### Complete blood counts, serum chemistries and systemic inflammation parameters

Complete blood counts and serum chemistries were measured with a Hemavet automated analyzer (Drew Scientific) and VET Test automated analyzer (IDEXX), respectively. Serum myeloperoxidase (MPO) activity was measured using a specific modified o-dianisidine-assay containing 4-aminobenzoic acid hydrazide as a potent and specific inhibitor of the MPO[24]. Cytokine concentrations, including IL-1β, IL-6, IL-8, IL-10, TNF-α and IFN-γ, were measured with commercial enzyme-linked immunosorbent assay (ELISA) kits reactive to porcine cytokines (R&D Systems).

### Assessment of leukocyte activation

FITC-conjugated anti-porcine CD11b antibody (SeroTec) was added to pre-chilled peripheral blood. Red cells were then lysed and the leukocytes fixed by addition of Bectin-Dickenson's FACS lysing solution. Cells were collected by centrifugation and resuspended for flow-cytometry analysis. CD11b expression was quantitatively assessed as mean fluorescent intensity (MFI) with an Accuri flow cytometer.

Peripheral blood mononuclear cells (PB-MCs) were isolated from venous blood samples of the animals. Mononuclear cells were isolated using standard Ficoll-Hypaque gradient technique (20). These cells were then incubated for 24 hrs in culture plates containing RPMI-1640 medium supplemented with antibiotics in the absence (unstimulated) an in the presence of 1 µg/mL lipopolysaccharide (LPS, stimulated). The supernatants were collected and cytokine concentrations measured. The ratio of stimulated to unstimulated cytokine concentrations in the supernatants was then calculated.

### Lung histology and immunohistochemistry

Lung samples were harvested post-mortem from septic pigs treated under SCD-citrate or SCD-heparin conditions. Two random sections from each of the 5 lobes of the lungs were processed for cryosections. Frozen lung samples were cut at 5-µm thickness and fixed with 4% paraformaldehyde on ice for 10 minutes. Tissues were stained with hematoxylin and eosin for light microscopic examination, or for CD11b evaluation; nonspecific adsorption was minimized by incubating the section in goat serum in PBS for 1 hour. The sections were then incubated with primary anti-CD11b at recommended dilution for 1 hour at room temperature, followed by incubation with 1:200 anti-mouse IgG Alexafluor594 conjugate at room temperature for 30 minutes, then nuclei counterstained with DAPI. ImageJ software [25] was used to quantitate the percent CD11b-positive area in random 10x images taken with fixed capture settings and then normalized to cell number by percent DAPI-positive area in the same picture. The results are expressed as a ratio of percent CD11b-positive area by percent DAPI-positive area.

### Cell elution of SCD cartridges

At the end of the experiment, blood was returned to the pig by perfusion with replacement fluid. The circuit was disconnected from the pig and replacement fluid flushed continuously through the SCD extracapillary space (ECS) until perfusate fluid was free of visible blood. Replacement fluid was drained from lumen and ECS of the cartridge and the cartridge was fixed for histologic processing [26] or exchanged with a proprietary stabilization buffer containing a calcium chelating agent. Adherent cells were then mechanically removed from the SCD eluent for analysis. To ensure that all cells adherent to the device were eluted, several cartridges were digested after elution with a DNA isolation buffer (SDS and proteinase K) and the DNA isolated and quantified. The DNA extracted from cells remaining in the cartridge after elution, on average, represented less than 5 percent of the total number of cells recovered from the cartridge.

### *In Vitro* assessment of leukocyte interaction with the SCD membrane

A custom microscopic flow chamber system was set up to enable microscopic analysis of leukocyte interaction with a therapeutic membrane under investigation. The flow chamber consisted of a polycarbonate housing with an inlet and outlet for perfusion. A flat sheet polysulfone membrane was affixed to the block with a polycarbonate gasket to direct the shear flow. Thickness of the gasket (100 µm), length (2 cm), and the width of the channel (1.5 mm) determined the volume of the flow chamber. Microscopic imaging of the leukocyte/membrane interaction was accomplished through an optical window of cover glass affixed to the bottom of the polycarbonate block. This microscopic flow chamber was used with isolated blood, or purified leukocytes.

Isolated blood is prone to activation with excessive handling; therefore, 5 mL of fresh heparinized porcine blood was prepared for custom flow chamber evaluation with minimal manipulation. Briefly, leukocytes were fluorescently labeled using 50 µg/ml of Hoechst 33342, a membrane permeable, nuclear intercalating dye. The effect of activation of leukocytes within whole blood was assessed by adding 1 µg/ml lipopolysaccharide (LPS) directly to blood samples. Similarly, 125 µL of anticoagulant citrate dextrose solution USP Formula A (Baxter, Deerfield, IL) was added to isolated blood and ioinized calcium levels were measured prior to microscopic flow analysis with i-stat EG-7+ cartridges. Blood passed through the flow chamber at a rate of 20 µL/min. with calculated shear forces between 1–10 dynes/cm^2^. For each isolated blood sample, sequences were acquired in triplicate.

Microscopic analysis of cell capture events was accomplished either using a Zeiss Axiovert 200 M or Axio-Observer epifluorescence microscope equipped with a microscope stage-top incubator to control environmental temperature and CO_2_ content. Fluorescence images were acquired with either a Zeiss MRm3 or an Icc1 camera at a frequency of 1 frame/second for 5 minutes, allowing for analysis of the leukocyte/membrane interaction, and at 1 frame/minute for 1 hr sequences to assess long term attachment. Frame by frame evaluation of rolling, attachment and detachment of leukocytes was carried out to determine the total number and duration of these phenomena. An attachment event was defined when a leukocyte appeared in the same location for multiple frames within a sequence. Detachment was defined as release events associated with previously defined attached leukocytes. Rolling events were defined by identifying the same leukocyte in multiple sequence frames within a sequence where the leukocyte was not in same exact location, but in close proximity to the prior location, and was moving slower than the fluid flow.

### Assessment of *in vitro* leukocyte activation

Heparinized whole blood was added to microfuge tubes prepared with and without lipopolysaccharide (LPS) (10 µg/mL) or formyl-methionyl-leucyl-phenylalanine (fMLF, 50 nM) and/or anticoagulant citrate dextrose solution (ACD) with gentle mixing [27], [28]. Measurement of various components utilized commercially available ELISA kits from R+D Systems (IL-6, IL-8, IL-10), from Bender MedSystems (elastase) and EMD chemical (lactoferrin); iCa levels for the (+) citrate and (−) citrate conditions were measured using an I-STAT reader and confirmed to be ≤0.25 mM and 1.25 mM, respectively. Samples were incubated for various times at 37°C and 5% CO_2_. Plasma samples were collected and analyzed. CD11b activation was measured using an FITC-conjugated mouse anti-human antibody (AbD Serotec) and evaluated on an Accuri C6 flow cytometer.

### Statistics

Group comparisons at multiple time points utilized ANOVA with repeated measures. Otherwise, comparisons between groups used Students' t test, paired or unpaired, as appropriate. Statistical significance was defined as p<0.05.

## Results

### Cardiovascular parameters

**Figure 2:**
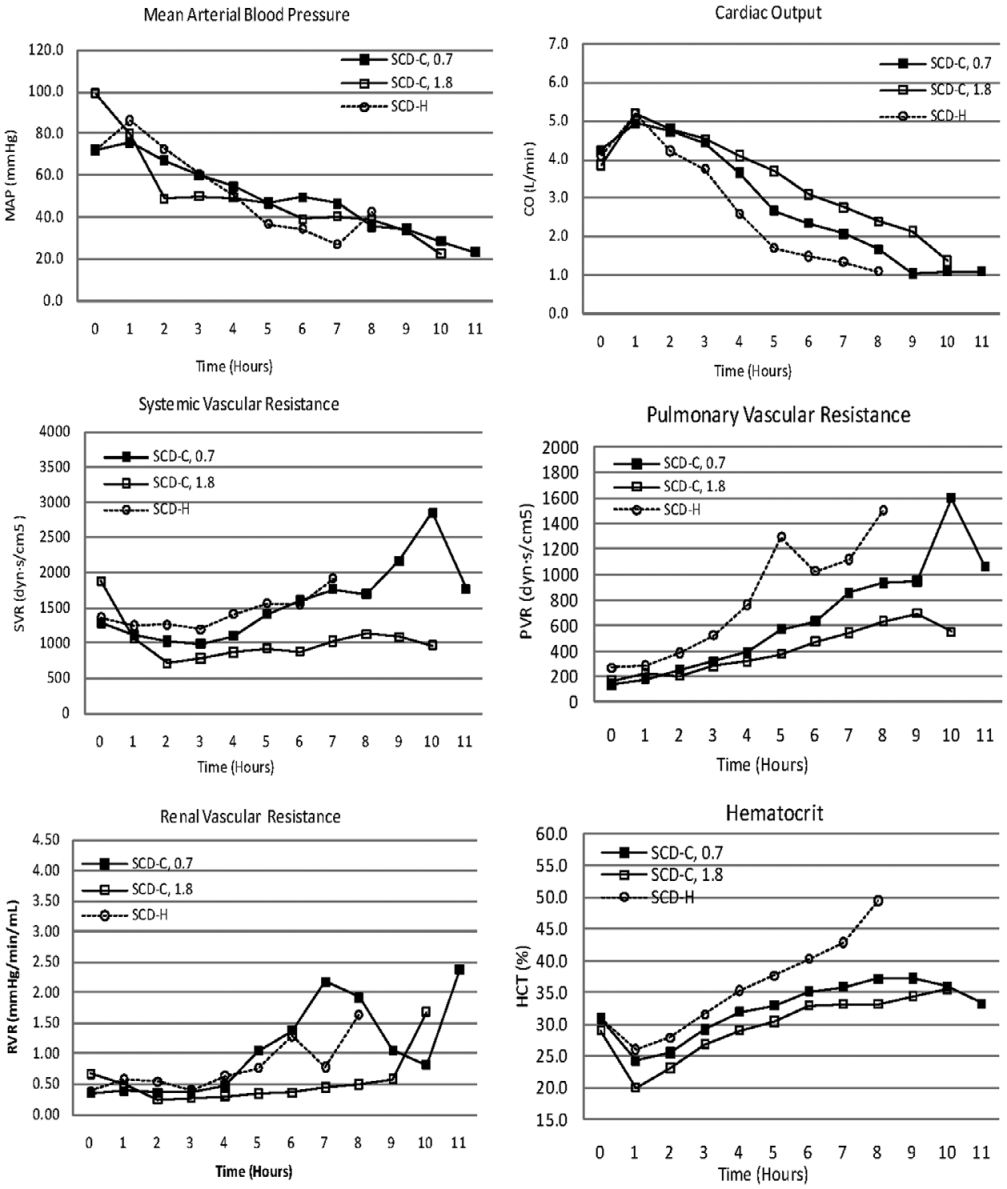
Cardiovascular parameters of SCD groups. Statistical comparisons in text.

The intraperitoneal administration of high-dose gram-negative *E. coli* bacteria in this porcine study produced a rapid, profound and ultimately fatal decline in arterial blood pressures (Table 1 and Fig. 2). These declines occurred soon after bacteria administration and were progressive with modest volume resuscitation but without vasopressor or inotropic support. Although mean arterial blood pressure was similar in the groups, cardiac output (CO) was significantly higher (p<0.02) in the SCD-citrate versus SCD-heparin groups (Fig. 2). This increase in CO was not due to differences in left ventricular filling pressures, since pulmonary capillary wedge pressures were similar in both groups, but was associated with a lower level of systemic vascular resistance of the SCD-citrate group (p<0.03, Fig. 2). Pulmonary vascular resistance (PVR, Fig. 2, p<0.001) was less in the SCD-citrate groups compared to the SCD-heparin group. Despite no consistent difference in RBF in the SCD-H versus SCD-C, 0.7 group, the SCD-C, 1.8 group, had a significant (p<0.02) reduction in RVR at the later time points compared to SCD-H. As a quantitative measure of systemic capillary leak process activated with bacterial sepsis, the changes in hematocrit were assessed. Hematocrit values progressively increased during the course of the experiment in the SCD-H group compared to a plateauing of values after 6 hours in the SCD-C group (Fig. 2, p<0.02). Of importance, the SCD displayed a dose effect with the SCD-C, 1.8 group, having significantly better preservation of cardiac output in the later time periods compared to SCD-C, 0.7, along with significantly lower SVR, PVR, and RVR. The Con-citrate animals had similar cardiovascular changes compared to the SCD-H group (Table 1). The non-septic (NS)-control animals had stable cardiovascular parameters through 8 hours, at which time the experiment was terminated.

**Table 1:** Cardiovascular parameters.

Parameter	0	1	2	3	4	5	6	7	8	9	10	11
**Cardiac output, L/min**												
** SCD-Citrate 0.7 m^2^**	4.3±0.3	4.9±0.2	4.7±0.2	4.4±0.3	3.7±0.2	2.7±0.3	2.3±0.2	2.1±0.3	1.7±0.1	1.0±0.3	1.1±0.1	1.1±0.1
** SCD-Citrate 1.8 m^2^**	3.9±0.8	5.2±0.6	4.8±0.3	4.5±0.4	4.1±0.5	3.7±0.5	3.1±0.2	2.8±0.2	2.4±0.3	2.1±0.4	1.4±0.2	
**SCD-Heparin**	4.1±0.3	5.2±0.2	4.2±0.3	3.8±0.2	2.6±0.2	1.7±0.2	1.5±0.2	1.3±0.2	1.1			
**Con-Citrate**	4.5±0.3	4.7±0.5	5.2±1.2	3.6±0.5	3.8±0.5	2.6±0.4	1.5±0.3	1				
**Systolic blood pressure, mmHg**												
** SCD-Citrate 0.7 m^2^**	96.9±5.7	99.9±2.2	94.5±3.2	88.9±4.4	80.3±4.1	69.7±6.5	69.5±7.0	68.0±6.5	55.0±8.7	45.8±5.1	53.5±0.5	36.5±8.5
** SCD-Citrate 1.8 m^2^**	118.7±29.2	98.7±9.7	65.7±4.4	70.3±4.1	69.0±5.1	67.0±4.6	59.3±4.5	60.7±8.7	61.7±8.1	51.0±4.5	33.3±7.9	
**SCD-Heparin**	96.6±4.7	104.9±4.8	94.4±6.5	88.0±4.4	76.4±6.3	58.4±4.4	52.4±8.4	41.0±12.1	55			
**Con-Citrate**	87.3±1.8	103.0±11.4	77.3±4.2	69.0±3.2	74.7±13.7	51.7±4.9	30.0±20.0					
**Diastolic blood pressure, mmHg**												
** SCD-Citrate 0.7 m^2^**	60.5±4.6	64.5±2.9	54.0±4.7	45.5±4.4	42.1±4.7	39.7±4.8	39.9±4.8	36.1±3.4	26.3±3.2	26.5±4.7	32.5±4.5	19.5±2.5
** SCD-Citrate 1.8 m^2^**	89.3±25.9	70.0±6.1	40.3±6.6	40.0±1.0	39.3±1.2	36.7±1.2	29.0±0.6	30.3±1.8	27.3±1.9	25.0±2.9	17.0±3.5	
**SCD-Heparin**	61.4±3.3	75.6±4.5	61.7±6.6	48.3±3.4	38.6±3.6	27.6±3.4	26.1±5.1	24.0±7.3	36.5			
**Con-Citrate**	53.3±2.0	71.7±6.3	50.3±4.5	42.7±1.5	48.3±12.9	31.0±2.1	20.0±10.0					
**Mean arterial pressure, mmHg**												
** SCD-Citrate 0.7 m^2^**	72.2±4.8	75.8±2.6	67.2±4.1	59.9±4.2	54.8±3.9	47.1±6.4	49.5±4.5	46.5±3.7	35.7±4.9	34.3±5.3	28.4±10.1	23.3±2.7
** SCD-Citrate 1.8 m^2^**	99.1±27	79.6±7.3	48.8±5.8	50.1±1.5	49.2±2.5	46.8±2.1	39.1±1.6	40.4±3.9	38.8±3.9	33.7±3.3	22.4±4.9	
**SCD-Heparin**	72.0±3.3	86.1±4.4	72.6±6.5	60.6±3.1	50.3±4.4	36.5±3.6	34.3±6.3	26.8±8.6	42.7±0.3			
**Con-Citrate**	64.7±1.7	82.1±8.0	59.3±4.1	51.4±1.1	44.5±0.5	37.9±2.9	23.3±13.3					
**Systemic vascular resistance, dyn·s/cm^5^**												
** SCD-Citrate 0.7 m^2^**	1288±119	1119±61	1027±73	994±72	1101±64	1414±111	1601±143	1767±204	1701±179	2170±183	2856±722	1776±336
** SCD-Citrate 1.8 m^2^**	1881±152	1073±23	710±143	784±59	874±114	926±131	884±59	1028±139	1134±186	1088±87	971	
**SCD-Heparin**	1371±137	1250±120	1268±110	1200±58	1412±75	1567±140	1552±242	1918±533				
**Con-Citrate**	1034±111	1149±94	1067±72	976±96	1174±103	1375±343	1274					
**Pulmonary vascular resistance, dyn·s/cm^5^**												
** SCD-Citrate 0.7 m^2^**	141±17	180±25	255±33	321±47	393±78	573±118	632±97	859±145	935±131	948±343	1602±242	1067±133
** SCD-Citrate 1.8 m^2^**	164±13	228±83	207±86	281±63	317±55	377±55	475±61	543±54	634±49	694±58	552	
**SCD-Heparin**	268±102	287±51	384±46	525±58	763±76	1293±243	1024±198	1121±291	1504			
**Con-Citrate**	147±18	122±17	404±177	602±83	525±151	982±248	1199±14					
**Pulmonary capillary wedge pressure, mmHg**												
** SCD-Citrate 0.7 m^2^**	7.8±0.7	8.5±0.9	8.3±1.0	7.0±1.1	7.2±1.1	7.2±1.1	5.9±0.9	5.9±0.8	4.9±1.0	6.8±2.1	5.0±2.6	3.5
** SCD-Citrate 1.8 m^2^**	8.3±0.9	11.3±2.4	10.7±3.7	7.3±1.2	6.3±0.9	5.7±0.9	6.0±0.6	6.3±0.7	6.3±0.7	6.0±0.6	12.0±5.5	
**SCD-Heparin**	7.0±0.8	8.5±1.2	7.2±0.8	6.6±0.7	7.3±1.4	6.3±1.0	5.7±1.0	6.8±1.0	5.5			
**Con-Citrate**	7.7±1.2	10.7±0.9	9.0±1.5	7.3±1.3	6.3±0.3	6.3±0.3	8.5±1.5					
**Renal arterial blood flow, mL/min**												
** SCD-Citrate 0.7 m^2^**	197.4±16.9	183.7±12.8	193.4±25.5	173.2±23.4	125.1±18.2	79.9±18.0	69.3±17.9	48.5±14.7	37.1±11.8	37.0±13.9	47.5±12.5	13.5±8.5
** SCD-Citrate 1.8 m^2^**	152.0±15.5	141.0±2.3	170.7±31.5	173.3±33.5	153.0±23.9	131.3±26.9	103.0±23.5	83.0±13.1	67.3±8.2	49.7±9.2	30.5±24.5	
**SCD-Heparin**	207.0±22.8	155.2±15.7	152.0±21.7	148.5±18.8	111.8±21.4	53.4±13.6	37.6±13.8	45.8±20.1	24			
**Con-Citrate**	200.3±19.5	157.3±38.1	184.3±63.0	183.0±48.3	138.0±17.7	69.0±24.0	19.0±19.0					
**Renal vascular resistance, mmHg/min/mL**												
** SCD-Citrate 0.7 m^2^**	0.39±0.03	.037±.0.6	0.37±0.05	0.48±0.07	1.05±0.29	1.37±0.44	2.18±0.63	1.93±0.72	1.05±0.31	0.82±0.37	2.38±1.56	
** SCD-Citrate 1.8 m^2^**	0.67±0.27	0.49±0.06	0.25±0.05	0.28±0.07	0.30±0.05	0.35±0.08	0.44±0.09	0.50±0.08	0.59±0.07	1.69±1.14		
**SCD-Heparin**	0.39±0.08	0.58±0.08	0.55±0.11	0.41±0.04	0.63±0.20	0.77±0.16	1.30±0.37	0.78±0.23	1.64±0.30			
**Con-Citrate**	0.30±0.02	0.52±0.12	0.33±0.08	0.26±0.05	0.28±0.04	0.67±0.31	0.75					

**Figure 3:**
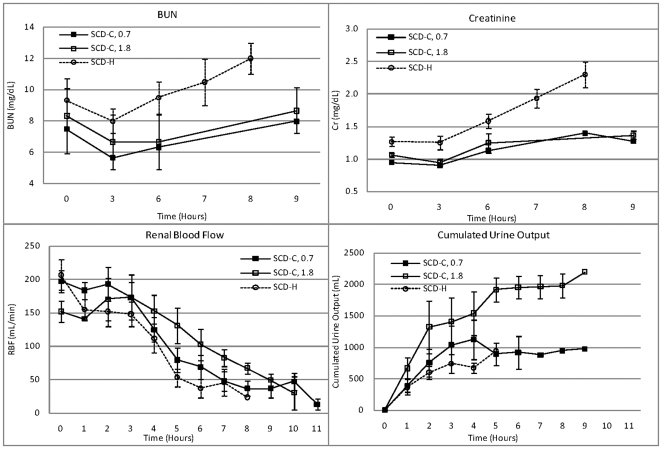
Renal function parameters in SCD groups. Statistical comparisons in text.

Specific detailed evaluation focused on renal parameters, since clinical testing has been and will be accomplished in acute renal failure patients in which the SCD can be easily incorporated into the extracorporeal blood circuit required for continuous renal replacement therapy (CRRT). In this regard, as shown in Figure 3, the degree of renal dysfunction in the SCD-C groups were much less than the SCD-H groups as reflected in BUN (p<0.02) and serum creatinine levels (p = 0.007). Renal blood flow was much better preserved in the SCD-C, 1.8 m^2^, group compared to SCD-H (p<0.05) as well as a greater urine output (p<0.05).

**Figure 4:**
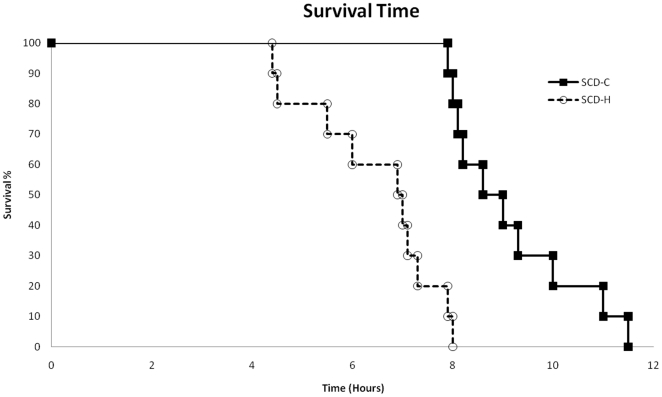
Survival curves in SCD groups.

These differences in cardiovascular and hemodynamic parameters in the two groups were associated with a significantly longer survival time in the SCD-C group compared to the SCD-H group (Figure 4). The citrate-treated animals survived 8.8±0.4 hours compared to 6.4±0.3 hours for the SCD-H animals (p = 0.0002). Of note, the SCD-C, 1.8 group, had survival times of 11.5, 10, and 9.5 hours. The survival curves of the two groups are displayed in Figure 3. The key component of the improved cardiovascular and organ function parameters in these experiments was the combination of both the SCD and citrate. The Con-citrate group of animals treated with a single hemofilter cartridge with blood perfusion through the hollow fiber lumens with citrate anticoagulation but without the SCD demonstrated similar cardiovascular parameters as the SCD-H group, with survival time of 6.5±0.5 hours (7, 6, and 5.5 hours for three animals). Thus, both the SCD and the citrate anticoagulation protocol were required to provide a survival advantage.

### Leukocyte parameters

**Figure 5:**
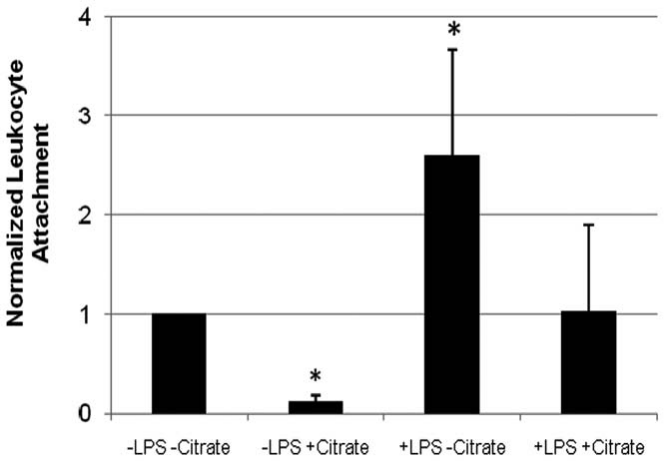
Leukocyte attachment to polysulfone membranes during shear flow in custom flow chambers. All values are reported as average normalized values to -LPS -Citrate ± Standard Error. (N = 3, p<0.05).

To assess the interactions of leukocytes and the SCD polysulfone membranes, a customized flow chamber with videomicroscopy was set up. The addition of citrate decreased ionized calcium levels of tested blood to 0.32±0.05 mmol/L, while ionized calcium in the normal blood samples was 1.32±0.05 mmol/L. Analysis of leukocyte attachment events confirms that LPS activation of the leukocytes in the absence of citrate significantly increases leukocyte attachment to polysulfone membranes during shear flow (p<0.05, Fig. 5). In citrate treated, low ionized calcium flow chambers, a statistically significant decrease in leukocyte attachment was observed (p<0.05), suggesting that leukocyte adhesion to polysulfone membranes may be ionized calcium dependent. These results are consistent with *ex vivo* data in the severe sepsis porcine model, in which citrate treated membrane cartridges have fewer adhered leukocytes at the end of studies, as assessed by elution of the cartridges (see below). In addition, preliminary analysis of 1 hour sequences demonstrated far fewer persistent leukocyte adhesion events for +LPS +Citrate isolated blood compared to +LPS -Citrate isolated blood; however, there was an observed increase in +LPS +Citrate rolling events. This suggests a catch and release phenomena when leukocytes interact with the polysulfone membrane in the presence of citrate.

Further experiments were carried out to assess the effect of citrate and a reduction of low iCa on leukocyte activity. An *in vitro* whole blood assay system was established [27], [28] to assess the effect of citrate-promoted reductions in blood iCa on leukocyte cytokine production (IL-6, IL-8, IL-10) and release of preformed inflammatory proteins from neutrophil exocytotic vesicles (lactoferrin, elastase). As demonstrated in Table 2, citrate-promoted declines in blood iCa levels were associated with diminution in both basal and stimulated (LPS, fMLF) promoted release of cytokines and exocytotic proteins.

**Table 2:** Effect of citrate on leukocyte activation parameters.

Baseline	IL-6(ng/mL)n = 7	IL-8(ng/mL)n = 5	IL-10(ng/mL)n = 4	Lactoferrin(mg/mL)n = 4	Elastase(mg/mL)n = 5	CD11b(MFI ×10^3^)n = 3
**Heparin**	0.18±0.04	0.0±0	0.11±0.07	8.47±1.54	2.73±0.29	22.55±1.06
**Citrate**	0.38±0.15	0.59±1.51	0.01±0.01	1.67±0.29*	0.94±0.14§	7. 32±0.47§
**Stimulated** **(LPS, fMLF)**						
**Heparin**	65.42±19.77	34.18±6.66	3.74±0.94	12.42±1.08	4.52±0.54	53.43±3.12
**Citrate**	28.99±7.60*	3.45±2.30†	2.06±0.84†	3.43±0.18§	0.91±0.28**	28.72±2.95§

*p<0.05;

†p<0.02;

**p<0.005;

§p<0.002, as determined with paired t-test between heparin and citrate groups.

**Figure 6:**
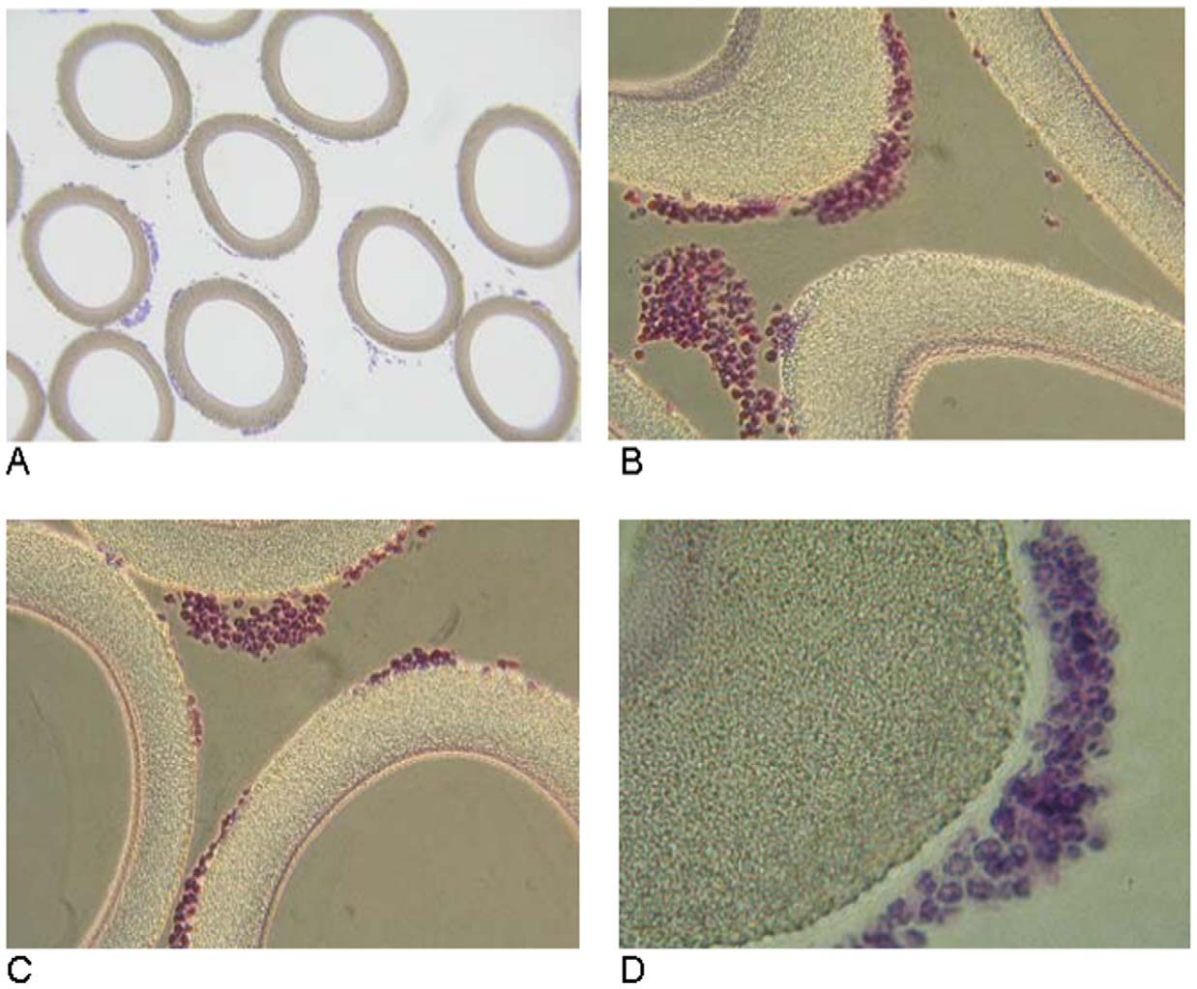
Leukocyte adherence to outer surfaces of hollow fibers. Light micrographs stained with H&E from three animals show leukocyte adherence to surfaces of the SCD. Panel A: Low-power micrograph showing adherent cells around each hollow fiber (160×). Panels B and C: Higher power micrographs demonstrating cluster of leukocytes along the outer surface of hollow fibers (400×). Panel D: High-power micrograph displaying predominant polymorphonuclear cells along with mononuclear cells in the adherent cell clusters (1600×).

To assess the sequestration of activated leukocytes along the SCD membranes, the SCD cartridges were processed for histological evaluation at the conclusion of the sepsis-induced fatality study. The light microscopy findings in Figure 6 clearly depict leukocyte attachment and aggregation along the outer surface of the SCD membranes. To determine the amount and type of adherent leukocytes in the SCD, the devices were processed and cells eluted off the membrane at the end of treatment period. The number of white blood cells (WBCs) eluted off the SCD-heparin and SCD-citrate, 0.7, devices were 6.44±3.4×10^8^ and 1.72±1.20×10^8^ cells (p<0.05), respectively, suggesting that citrate anticoagulation promoted a higher degree of detachment events during the treatment protocol. The distributions of eluted cells were 79±5% neutrophils and 21±4% monocytes in SCD-heparin vs. 55±4 and 30±5% SCD-citrate, 0.7 groups. Of note, the 1.8 SCD-C group had even fewer adherent cells averaging 1.88±1.21×10^7^, a tenfold lesser number than the 0.7 groups, reflective of the low circulating systemic white counts in the later stages of the experiments in the 1.8 group (see below). Activated leukocytes predominated this sequestration phenomenon, since an average 8×10^6^ cells eluted from the cartridges of non-septic control animals with SCD (n = 2). Of note, the C-citrate group had less than 2×10^4^ cells eluted from lumens of the cartridges with luminal blood perfusion.

**Figure 7:**
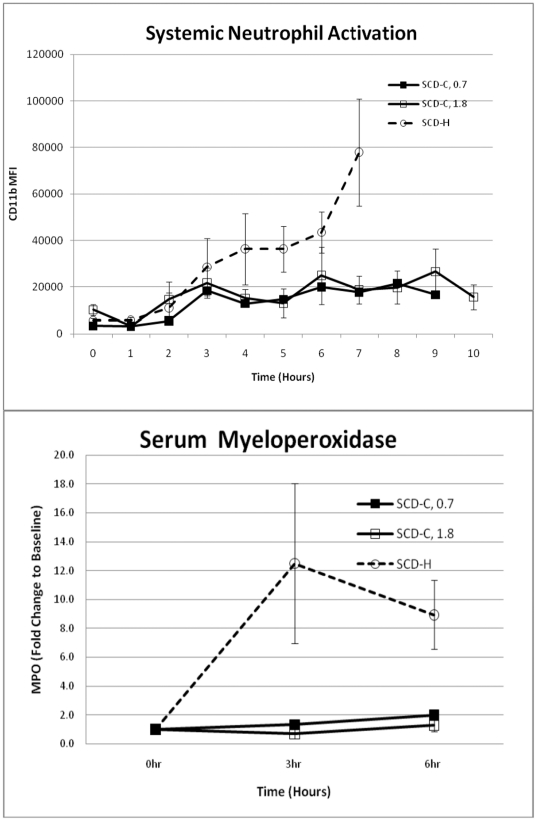
Serum MPO levels (top panel) and circulating leukocyte CD11b levels (bottom panel) in SCD groups.

To further test the hypothesis that the SCD with citrate anticoagulation can influence the activation state of circulating leukocytes, a variety of biomarkers were assessed in the animals during the course of the experimental protocol. Activated neutrophils release various enzymes in response to invading microbes or tissue injury to initiate tissue repair. Since the dominant enzyme released from neutrophil granules is MPO [29], blood levels of MPO reflect the level of activation of neutrophils in the body. Plasma MPO levels with SCD-citrate were significantly lower compared with SCD-heparin (p = 0.01 at hour 3), averaging 82.4±21.0 and 307±102 ng/mL, as well as well as at 6 hours (p<0.005, Fig. 7), respectively. Serum cytokine levels, including IL-1β, IL-6, IL-8, IL-10, TNF-α and IFN-γ, were not significantly different between SCD-heparin and SCD-citrate groups, although IL-1β and IL-8 trended higher in the SCD-heparin compared to the SCD-citrate group.

The level of neutrophil activation was quantified by measuring the amount of CD11b expression on circulating neutrophils. CD11b is a membrane protein involved in adherence of leukocytes to activated endothelium as a first step to exiting the circulation to a site of inflammation [30]. As detailed in Figure 7, the MFI of neutrophils in the systemic circulation was dramatically increased during the treatment time course in the SCD-heparin group compared to the SCD-citrate group (p = 0.03).

To further assess the effect of SCD-C on the innate immunologic system, PBMCs were isolated and assessed for cytokine release. At baseline prior to sepsis induction, PBMC release of TNF-α and IL-8 were 2.1±1.8 and 6.5±2.8 pg/10^6^ cells in response to LPS in the SCD-H group, respectively; in the SCD-C group, the release was 5.1±0.9 and 18.7±8.1, respectively. At 6 hours of sepsis, PBMC release of TNF- α and IL-8 in response to LPS was significantly lower in the SCD-C versus SCD-H groups (p<0.05), averaging 14±2 and 24±12 percent of baseline values for TNF- α and IL-8 in SCD-C animals compared to 63±10 and 108±19 percent of baseline levels in the SCD-H group. PBMC release of IL-6 was not different between groups.

**Figure 8:**
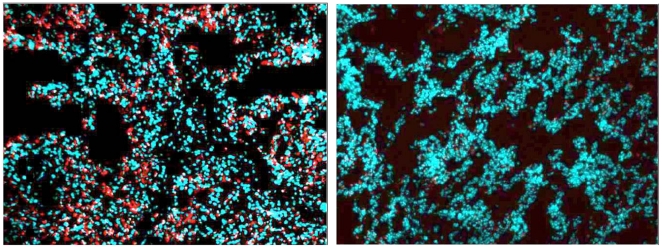
Neutrophil aggregation in lung tissue. Neutrophil aggregation was evaluated in cryosections of lung tissue prepared postmortem from septic animals treated with SCD–heparin (left) or SCD–citrate (right). CD11b was detected with Alexafluor 594, nuclei detected with DAPI and 10× images taken with the appropriate filter sets. Image J software was used to quantitate the percent of positive area in each CD11b picture and then normalized with DAPI percent area.

Since previous studies in animal models of sepsis have reported that the lung is the first organ target for activated leukocyte sequestration and infiltration after endotoxemia or sepsis [14], [15], we evaluated the effect of SCD and citrate anticoagulation on the sequestration of activated leukocytes in lung tissue compared to SCD-heparin animals. As demonstrated in Figure 8, a substantive decrease in CD11b-labeled cells was observed in the SCD-C group compared to the SCD-H animals. Careful histomorphometric analysis of 4 animals in the SCD-C group versus 5 animals in the SCD-H group demonstrated ratios of CD11b positive area to DAPI- positive area of 0.114±0.21 versus 0.334±0.052 (p = 0.007), respectively.

**Figure 9:**
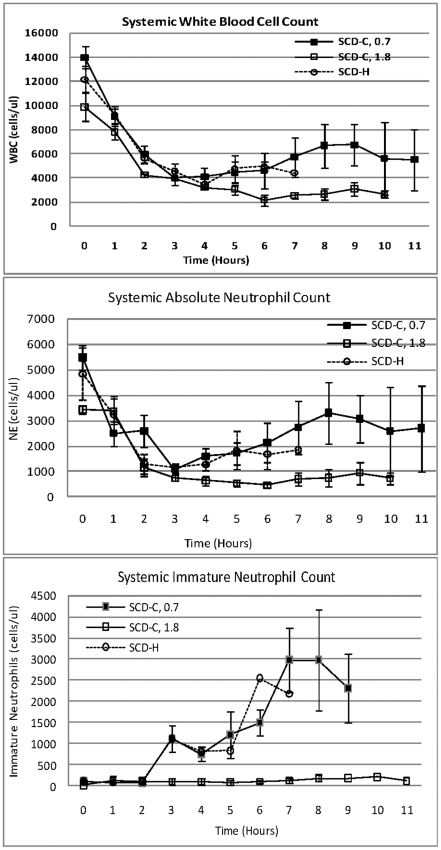
Time course of circulating white blood counts; total absolute neutrophil and immature neutrophil cell counts in various SCD groups.

To determine the kinetics of the circulating pool of leukocytes in the SCD-H and SCD-C groups, the absolute WBC and neutrophil counts were assessed (Fig. 9). Both groups reached a nadir of 1125±240 and 1094±166 neutrophils/mm^3^ at hour 3 in the heparin and citrate groups, respectively. These groups did not reach absolute neutropenia (defined as counts below 500) due to an increase in immature neutrophils from bone marrow, as assessed by manual examination of blood smears, beginning at 3 hours in both groups. Of note, the SCD-C, 1.8, group had a persistent low neutrophil count reaching a nadir of 457±77 at hour 6 due to a markedly diminished release of immature neutrophils from the bone marrow. The Con-citrate group had a similar decline and rebound of leukocyte counts, whereas the NS-control animals tended to have neutrophilia, with neutrophil counts rising from approximately 4,000 to 14,000 over the 8-hour evaluation period.

## Discussion

Sepsis continues to be a complex and therapeutically challenging clinical disease state. Sepsis is the leading cause of death in critically ill patients in the United States [1], [2]. The sepsis syndrome has been recognized as the clinical manifestation of an excessive host response to infection, or the systemic inflammatory response syndrome (SIRS). The signs and symptoms of SIRS are not a direct result of the infecting pathogens but are due to the dysregulation of the host's innate immunologic system, with activation of endothelial cells, leukocytes, and platelets [6], [7], [8]. The cardiovascular instability of SIRS with hypovolemia and hypotension is a consequence of leukocyte and endothelial activation resulting in increased vascular permeability, fluid leakage into the interstitium, and redistribution of intravascular fluid volume into interstitial spaces and generalized edema [7], [11]. The sequestration and infiltration of leukocytes into lung tissue promotes respiratory dysfunction, often progressing to ARDS [14], [15]. Sequestration and aggregation of activated cellular elements of the blood system in the renal microvasculature promotes renal dysfunction, AKI and ARF [12], [13].

Neutrophils are the initial and cardinal cellular effectors of the innate immunologic response. Because of the nonselectivity in their targets, neutrophils are highly effective in eradicating invading microbes and responding to tissue injury but with substantial collateral damage to local tissue. Controlling the levels of activation of circulating leukocytes during SIRS may be an effective means of treating sepsis in conjunction with antibiotics, fluid resuscitation, and artificial organ support. This modulation, however, must allow adequate neutrophil numbers and activation required for host defense but not excessive numbers and activation to promote multiorgan dysfunction. This modulation is emphasized since absolute neutropenia in clinical situations has been clearly shown to result in high susceptibility for infection [9], [10], yet neutrophil depletion, depending upon degree and timing after sepsis initiation, has been shown to ameliorate multiorgan dysfunction and fatal consequences [31], [32].

The manner in which an advanced membrane device coupled with a commonly used anticoagulation solution containing citrate can influence the multiorgan effects of sepsis and impact survival times may provide insight into the critical pathways in which the innate immunologic response promotes multiorgan dysfunction and fatal consequences. In this regard, we have formulated an extracorporeal biomimetic membrane device (the SCD) which preferentially binds activated leukocytes and, with a regional citrate anticoagulant circuit, promotes a lower systemic leukocyte activation profile leading to a sustained, but nonexcessive, leukocyte activation process during a septic shock episode. The SCD was developed with a proprietary design using an extracorporeal circuit and a cartridge containing biocompatible polysulphone hollow fibers. The blood flow within the device was directed to the extracapillary space of this cartridge to achieve a blood velocity and shear stress approaching capillary hemodynamics to provide a surface for leukocyte adherence. The ability of this device to sequester activated leukocytes was demonstrated both *in vitro* with videomicroscopy of a custom designed shear chamber containing a polysulfone membrane and *ex vivo* during extracapillary perfusion in a porcine model of gram-negative septic shock. Elution of cells adherent to the SCD membranes recovered approximately 10^8^ to 10^9^ leukocytes, a log-fold greater than non-septic conditions. Ongoing studies with shear chambers with blood perfusion over flat plate polysulphone membranes have demonstrated significantly greater attachment events after activation of leukocytes with LPS.

A second component to this continuous extracorporeal therapy was combining the SCD with regional citrate anticoagulation protocols. Citrate infusion achieves effective anticoagulation by chelating calcium and removing iCa as a cofactor for the coagulation cascade [21], [22]. This decline in iCa also inhibits key leukocyte activation processes as well [19], [33], so that in combination with citrate regional anticoagulation the SCD becomes a selective cytopheretic inhibitory device. In this regard, the experiments presented in this report demonstrate that lowering iCa in whole blood inhibits the release of cytokines (IL-6, IL-8, and IL-10) and neutrophil exocytotic proteins (lactoferrin and elastase) as well as CD11b expression after stimulation with LPS or fMLF. The low-iCa environment, promoted with citrate during the SCD treatment, also appears to promote detachment of the bound and sequestered leukocytes since the number of leukocytes bound to the SCD membrane was approximately 10-fold less than that observed with SCD during systemic heparin anticoagulation. In addition, citrate treatment to lower iCa in blood promoted less persistent attachment events in the *in vitro* shear chambers with SCD membranes.

To assess the efficacy of the SCD in SIRS, a well-established large-animal model of septic shock was studied [20]. The SCD was incorporated into an extracorporeal blood circuit and pump system used for continuous renal replacement therapy (CRRT) in anticipation of the clinical evaluation of the SCD in ICU patients with AKI and MOF. This clinical disorder is an appropriate initial application since AKI initiates a pro-inflammatory SIRS process [34] and is treated with continuous extracorporeal therapy with well-established citrate anticoagulation protocols. Accordingly, the use of the SCD in an extracorporeal CRRT circuit was evaluated in this porcine model during both regional citrate and systemic heparin anticoagulation protocols. The group of animals treated with the SCD and citrate maintained better cardiovascular parameters compared to SCD-heparin animals after the initiation of *E-coli* sepsis. More prolonged preservation of arterial pressures and higher cardiac outputs were observed in the SCD-C versus the SCD-H groups due to lesser degrees of systemic capillary leak. Systemic, pulmonary, and renal resistances were lower in the SCD-C compared to the SCD-H groups. These improved cardiovascular parameters translated to nearly 40 percent longer survival times. These effects were observed only in those animals treated with the combination of SCD and citrate; animals treated without the SCD with citrate anticoagulation had similar cardiovascular and neutrophil activation parameters and survival times as the SCD-heparin group. A dose effect with SCD-C on cardiovascular parameters was also observed when the membrane surface area was increased from 0.7 to 1.8 m^2^.

These physiologic parameters were associated with significant effects on neutrophil activity in the systemic circulation. The level of plasma MPO, a key exocytotic enzyme in the neutrophils respiratory burst process [29], rose dramatically in the SCD-H animals to a level 10–12 times the baseline level at 3 and 6 hours after sepsis induction, whereas MPO levels were unchanged in the SCD-C animals. It should be noted that MPO can be released from the vascular endothelium by the administration of heparin [35], but at levels that would not significantly impact those reported in Fig. 7. The level of CD11b expression in the circulating pool of neutrophils, as determined by flow cytometry, was also significantly reduced in the SCD-C group compared to the SCD-H group. These differences in biomarkers of neutrophil activation had a direct correlate to organ involvement. Since the lungs are the initial site of neutrophil sequestration in SIRS [14], [15], the number of activated leukocytes in lung tissue was measured by careful immunohistochemical morphometrics. The degree of accumulation in lung tissue was significantly higher in the SCD-H group compared to the SCD-C group. This increase in leukocyte accumulation was reflected in significantly higher PVR in the heparin versus citrate SCD animals. Furthermore, the degree of renal dysfunction as measured with BUN and serum creatinine levels was substantially worse in the SCD-H versus the SCD-C groups. Since the SCD also sequesters circulating monocytes from the elution studies, PBMCs were isolated from SCD-C and SCD-H groups. At 6 hours after sepsis, the response to LPS induced IL-8 and TNF-α release were modulated downward in SCD-C animals versus SCD-H. These results demonstrate an immunomodulatory role of the SCD-C in the response to sepsis.

The WBC kinetics in these studies also provide insight into the manner in which SCD treatment may influence the leukocyte response to infection. The number of neutrophils sequestered in the SCD did not exceed more than 10^9^ cells, a small percentage of the circulating and marginated pool [8]. Furthermore, despite the decline in circulating neutrophil counts due to sequestration and infiltration of these cells in various body compartments, especially the peritoneal cavity, absolute neutropenia did not occur due to a release of immature neutrophils from the bone marrow. The magnitude of this bone marrow response was blunted with the larger SCD membrane, suggesting that SCD-C treatment may alter the kinetics of neutrophil apoptosis and bone marrow release of neutrophils. Ongoing studies are planned to evaluate this process in greater detail. Of interest is the finding that the number of leukocytes in the SCD cartridge during citrate anticoagulation was 10-fold less than in the heparin condition despite a greater exposure time to sepsis before cartridge elution. This finding suggests that the low-iCa environment may promote release of adherent activated leukocytes. The kinetics of this “catch and release” phenomenon was supported with ongoing studies utilizing *in vitro* shear chambers. Since a log less leukocytes are bound to the SCD membrane and substantially less white cells were sequestered in the lung under citrate versus heparin anticoagulation conditions, the SCD-citrate animals may have also been advantaged with more neutrophils available to respond locally to the peritoneal infection. These studies suggest that this novel device and approach may ameliorate the natural progression of SIRS due to immunomodulation that leads to improved cardiovascular stability, respiratory performance and renal function. This study, however, demonstrates a preventative therapeutic approach initiated at the onset of bacterial infection. The effect of this treatment protocol on pre-existing septic shock in a similar model remains undefined. Initial attempts to reverse developing septic shock in a porcine model 2 hours after *E.coli* administration and sepsis onset was not successful. Incorporation of the extracorporeal circuit 2 hours into the course of sepsis resulted in substantive afterload reduction in the unstable animal and rapid cardiovascular collapse, although the outcome may have been different if the animals were supported with pressors as is routine in clinical practice for this disease state.

The clinical evaluation of the SCD with citrate anticoagulation has been initiated in two exploratory clinical trials in ICU patients with ARF and multiorgan failure due to the ease of incorporating this device into the standard CRRT approach to these critically ill patients. These early exploratory clinical trials have demonstrated an excellent safety profile and compelling efficacy impact [26], [36]. Leukopenia and sustained thromobocytopenia were not observed in these initial exploratory clinical studies. Accelerated renal recovery with CRRT discontinuation and a 50 percent or greater relative improvement in survival rates has been observed. A pivotal FDA-approved IDE, multicenter, randomized control trial is currently underway to evaluate this innovative approach to this clinical disorder.

## References

[1] Angus DC, Linde-Zwirble WT, Lidicker J, Clermont G, Carcillo J (2001). Epidemiology of severe sepsis in the United States: analysis of incidence, outcome, and associated costs of care.. Crit Care Med.

[2] Dombrovskiy VY, Martin AA, Sunderram J, Paz HL (2007). Rapid increase in hospitalization and mortality rates for severe sepsis in the United States: a trend analysis from 1993 to 2003.. Crit Care Med.

[3] Vincent JL, Sakr Y, Sprung CL, Ranieri VM, Reinhart K (2006). Sepsis in European intensive care units: results of the SOAP study.. Crit Care Med.

[4] Beale R, Reinhart K, Brunkhorst FM, Dobb G, Levy M (2009). Promoting Global Research Excellence in Severe Sepsis (PROGRESS): lessons from an international sepsis registry.. Infection.

[5] Hotchkiss RS, Karl IE (2003). The pathophysiology and treatment of sepsis.. N Engl J Med.

[6] Brown KA, Brain SD, Pearson JD, Edgeworth JD, Lewis SM (2006). Neutrophils in development of multiple organ failure in sepsis.. Lancet.

[7] Schouten M, Wiersinga WJ, Levi M, van der Poll T (2008). Inflammation, endothelium, and coagulation in sepsis.. J Leukoc Biol.

[8] Marshall JC (2005). Neutrophils in the pathogenesis of sepsis.. Crit Care Med.

[9] Hughes WT, Armstrong D, Bodey GP, Brown AE, Edwards JE (1997). 1997 guidelines for the use of antimicrobial agents in neutropenic patients with unexplained fever. Infectious Diseases Society of America.. Clin Infect Dis.

[10] Boxer LA (2003). Neutrophil abnormalities.. Pediatr Rev.

[11] Aird WC (2003). The role of the endothelium in severe sepsis and multiple organ dysfunction syndrome.. Blood.

[12] Sutton TA, Fisher CJ, Molitoris BA (2002). Endothelial injury and dysfunction during the extension phase of acute renal failure.. Kidney Int.

[13] Sutton TA, Mang HE, Campos SB, Sandoval RM, Yoder MC (2003). Injury of the renal microvascular endothelium alters barrier function after ischemia.. Am J Physiol Renal Physiol.

[14] Welbourn CR, Young Y (1992). Endotoxin, septic shock and acute lung injury: neutrophils, macrophages and inflammatory mediators.. Br J Surg.

[15] Andonegui G, Zhou H, Bullard D, Kelly MM, Mullaly SC (2009). Mice that exclusively express TLR4 on endothelial cells can efficiently clear a lethal systemic Gram-negative bacterial infection.. J Clin Invest.

[16] Nelson S, Belknap SM, Carlson RW, Dale D, DeBoisblanc B (1998). A randomized controlled trial of filgrastim as an adjunct to antibiotics for treatment of hospitalized patients with community-acquired pneumonia. CAP Study Group.. J Infect Dis.

[17] Root RK, Lodato RF, Patrick W, Cade JF, Fotheringham N (2003). Multicenter, double-blind, placebo-controlled study of the use of filgrastim in patients hospitalized with pneumonia and severe sepsis.. Crit Care Med.

[18] Kaneider NC, Leger AJ, Kuliopulos A (2006). Therapeutic targeting of molecules involved in leukocyte-endothelial cell interactions.. FEBS J.

[19] Tintinger G, Steel HC, Anderson R (2005). Taming the neutrophil: calcium clearance and influx mechanisms as novel targets for pharmacological control.. Clin Exp Immunol.

[20] Humes HD, Buffington DA, Lou L, Abrishami S, Wang M (2003). Cell therapy with a tissue-engineered kidney reduces the multiple-organ consequences of septic shock.. Crit Care Med.

[21] Pinnick RV, Wiegmann TB, Diederich DA (1983). Regional citrate anticoagulation for hemodialysis in the patient at high risk for bleeding.. N Engl J Med.

[22] Lohr JW, Slusher S, Diederich D (1989). Safety of regional citrate hemodialysis in acute renal failure.. Am J Kidney Dis.

[23] Tobe SW, Aujla P, Walele AA, Oliver MJ, Naimark DM (2003). A novel regional citrate anticoagulation protocol for CRRT using only commercially available solutions.. J Crit Care.

[24] Fietz S, Bondzio A, Moschos A, Hertsch B, Einspanier R (2008). Measurement of equine myeloperoxidase (MPO) activity in synovial fluid by a modified MPO assay and evaluation of joint diseases - an initial case study.. Res Vet Sci.

[25] Abramoff MD, Magelhaes PH, Ram SJ (2004). Image Processing with ImageJ.. Biophotonics International.

[26] Humes HD, Sobota JT, Ding F, Song JH, The RAD Investigator Group (2010). A selective cytopheretic inhibitory device to treat the immunological dysregulation of acute and chronic renal failure.. Blood Purication.

[27] Damsgaard CT, Lauritzen L, Calder PC, Kjaer TM, Frokiaer H (2009). Whole-blood culture is a valid low-cost method to measure monocytic cytokines - a comparison of cytokine production in cultures of human whole-blood, mononuclear cells and monocytes.. J Immunol Methods.

[28] Wutzler S, Maier M, Lehnert M, Henrich D, Walcher F (2009). Suppression and recovery of LPS-stimulated monocyte activity after trauma is correlated with increasing injury severity: a prospective clinical study.. J Trauma.

[29] Klebanoff SJ (2005). Myeloperoxidase: friend and foe.. J Leukoc Biol.

[30] Fan ST, Edgington TS (1993). Integrin regulation of leukocyte inflammatory functions. CD11b/CD18 enhancement of the tumor necrosis factor-alpha responses of monocytes.. J Immunol.

[31] Hoesel LM, Neff TA, Neff SB, Younger JG, Olle EW (2005). Harmful and protective roles of neutrophils in sepsis.. Shock.

[32] Navarini AA, Lang KS, Verschoor A, Recher M, Zinkernagel AS (2009). Innate immune-induced depletion of bone marrow neutrophils aggravates systemic bacterial infections.. Proc Natl Acad Sci U S A.

[33] Mandeville JT, Maxfield FR (1996). Calcium and signal transduction in granulocytes.. Curr Opin Hematol.

[34] Simmons EM, Himmelfarb J, Sezer MT, Chertow GM, Mehta RL (2004). Plasma cytokine levels predict mortality in patients with acute renal failure.. Kidney Int.

[35] Baldus S, Rudolph V, Roiss M, Ito WD, Rudolph TK (2006). Heparins increase endothelial nitric oxide bioavailability by liberating vessel-immobilized myeloperoxidase.. Circulation.

[36] Ding F, Yevzlin AS, Xu ZY, Zhou U, Xie QH (2010). The Effects of a Novel Therapeutic Device on Acute Kidney Injury Outcomes in the Intensive Care Unit: A Pilot Study..

